# The Effect of Post-Emergence Application of Biostimulants and Soil Amendments in Maize Cultivation on the Growth and Yield of Plants

**DOI:** 10.3390/plants14091274

**Published:** 2025-04-22

**Authors:** Łukasz Sobiech, Monika Grzanka, Robert Idziak, Andrzej Blecharczyk

**Affiliations:** Department of Agronomy, Faculty of Agriculture, Horticulture and Biotechnology, Poznań University of Life Sciences, Wojska Polskiego 28 St., 60-637 Poznań, Poland; lukasz.sobiech@up.poznan.pl (Ł.S.); monika.grzanka@up.poznan.pl (M.G.); robert.idziak@up.poznan.pl (R.I.)

**Keywords:** *Bacillus* sp., humic acids, *Methylobacterium symbioticum*, *Rhizophagus* sp., soil moisture conditions

## Abstract

Maize is considered to be one of the most significant crops in the world. On a global scale, the appropriate yield level of food can largely affect food security. During cultivation, this plant is exposed to many adverse environmental factors, including water deficiency. Plant stress is reduced by applying appropriate biostimulants or soil amendments. This study tested the effectiveness of preparations based on *Rhizophagus irregularis*, humic acids, *Bacillus velezensis* + *Bacillus licheniformis* and *Methylobacterium symbioticum*. The aim of the project was to assess the effect of selected microorganisms and substances on the growth, yield, and physiological parameters of maize. The hypothesis assumed that the preparations selected for this study could improve the condition of the plants in various soil moisture conditions. All treatments were carried out post-emergence. The experiments were conducted in greenhouse conditions, where, in conditions of different level of soil moisture, optimal and water deficiency, the effect of the above-mentioned substances and microorganisms on the height, mass of plants, and plant chlorophyll fluorescence was determined. Chlorophyll, anthocyanin, and flavonol content were also measured. In two-year field studies, the effect of the same preparations on plant height, grain yield, thousand-grain weight, oil, protein, and starch content in the grain was determined. It was shown that appropriately selected biostimulants have a positive effect on plant growth, physiological parameters, and the yield of maize grain. The impact of preparations on the grain yield depended on the conditions that prevailed in the growing season.

## 1. Introduction

Yield of crops is influenced by a number of factors, including the selected variety, the intensity of diseases, pests, and weeds [1,2]. Habitat factors, such as water availability or temperature [3], are also important. Throughout the entire period of development, plants are exposed to many stresses, both biotic and abiotic. One of the most common stresses worldwide is drought [4]. Nitrogen deficiency may also be a factor limiting crop yields [5]. In many areas used for agriculture, the content of organic matter and the occurrence of microorganisms in the soil have been disturbed, among other things, by intensive cultivation, use of fertilizers, and pesticides [6]. In order to improve the condition of the plants, biostimulants and soil amendments are increasingly used. The research on such substances is largely conducted in the case of the most economically important plant species, including maize [7,8]. Maize can be used for various purposes—food production, animal feed, energy production, or even textile production [9,10]. The largest producers of this plant are the United States, China, and Brazil [11]. Research is being conducted on the possibility of improving the condition of this species by using various preparations based on bacteria, fungi, and humic acids [12]. Nowadays, more and more of such preparations are available. It is important to study the effect of biostimulants and soil amendments in different soil and climatic conditions to determine their influence on plant development in different regions of the world.

*Rhizophagus* sp. fungi (previously called *Glomus* sp.) are microorganisms that can form arbuscular mycorrhiza (AM) with plant roots [13]. The process involves the mycelium penetrating the interior of plant root cells [14]. The possibility of this type of mycorrhiza occurring is noted for 80% of land plant species in the world. In this symbiosis, the fungus obtains sugar from the roots of the host plant, and the plant has a better ability to absorb water and nutrients [15]. The use of mycorrhiza contributes to better plant nutrition and increased resistance to stress [16]. Moreover, during AM formation, various proteins are secreted, including glomalin, which has a positive effect on the chemical and physical properties of the soil [17]. The occurrence of mycorrhizal fungi varies depending on the habitat [18]. Therefore, they are commonly used in the form of preparations used in agriculture [19].

Humic acids consist of a mixture of weak organic acids, both aliphatic (carbon chains) and aromatic (carbon rings) [20]. They have a beneficial effect on the water capacity of the soil and the capacity for cation exchange, promote the availability of nutrients, and improve the growth of plant roots and shoots [21]. Due to their high carbon content, they have a beneficial effect on the development of soil microorganisms [22]. Humic substances contribute to reducing the sensitivity of plants to environmental stresses, and reduce the adverse effects of soil salinization [23,24]. They are classified as both biostimulants and soil amendments [25,26]. The multidirectional effects of humic substances mean that the global market for preparations based on them is constantly growing. They are available in various forms—liquid, powder, granule, and flakes [27,28].

The microorganisms increasingly used in agriculture are bacteria of the genus *Bacillus*. They are applied both to stimulate plant growth and to protect them against diseases and pests [29]. They are also applied to increase plant tolerance to abiotic and biotic stresses. Microorganisms of the genus *Bacillus* are classified as plant growth-promoting rhizobacteria (PGPR) [30,31]. One of the species used in agriculture is *Bacillus velezensis*. Different strains of this bacterium produce secondary metabolites, such as surfactin, bacillibactin, bacillomycin D, and fengycin, acting antagonistically towards pathogens [32]. Volatile compounds of these gram-positive bacteria have also been discovered, which activate induced systemic resistance (ISR) and support plant growth [33]. *Bacillus licheniformis* has significant properties for growth-promoting activity. It produces large amounts of physiologically active gibberellins [34], and has the ability to assimilate nitrogen and reduce this component to forms available to plants. *B. licheniformis* bacteria are considered to be capable of siderophore biosynthesis and phosphate solubilization [35].

Bacteria of the genus *Methylobacterium* spp., known as pink-pigmented methylotrophic bacteria (PPFMs), use methanol as a source of energy and carbon [36]. This substance is released during plant growth, which allows for their colonization by the above-mentioned microorganisms [37]. They can penetrate plants through stomata [38]. An example of bacteria belonging to this genus is *Methylobacterium symbioticum* [39]. The bacterium, thanks to the nitrogenase enzyme, converts atmospheric nitrogen (N_2_) into ammonium (NH_4_^+^) [40]. The transformed nitrogen is used for the synthesis of nucleic acids and plant proteins [41]. The use of this type of bacteria may reduce the use of mineral fertilizers. Searching for alternative sources of nitrogen is important due to the expected increase in environmental pollution with fertilizers, which is a consequence of the greater demand for food [42]. An additional motivation for research on the efficacy of bacteria is the unstable prices of nitrogen fertilizers caused, among others, by the increase in the price of natural gas, which is needed for their production [43].

The aim of this study is to evaluate the efficacy of biostimulants and soil amendments based on microorganisms and humic acids on the growth, physiological parameters, and yield level of maize.

## 2. Results

### 2.1. Greenhouse Research

Of the studied parameters, the moisture conditions of the soil have significantly contributed statistically to a decrease in the height of plants (by 4.7 cm), variable fluorescence (by 45.4 unit), maximum fluorescence (by 42.1 unit), and maximum quantum yield of PSII photochemistry (by 0.011 unit). They have also led to an increase in the value of minimal fluorescence (by 3.4 unit) and the content of anthocyanins (by 0.007 unit). The moisture conditions of the soil did not have a statistically significant effect on the content of chlorophyll and flavonols in the leaves (Table 1).

The use of preparations based on *Rhizophagus irregularis*, humic acids, and *Bacillus velezensis* + *Bacillus licheniformis* has contributed to a statistically significant increase in plant height. The lowest flavonol content was recorded after the use of humic acids and *Methylobacterium symbioticum*. The lowest value of anthocyanins was found for a combination in which *Methylobacterium symbioticum* was applied. Individual preparations did not have a statistically significant impact on the chlorophyll content and the parameters of fluorescence chlorophyll (Table 2).

**Figure 1:**
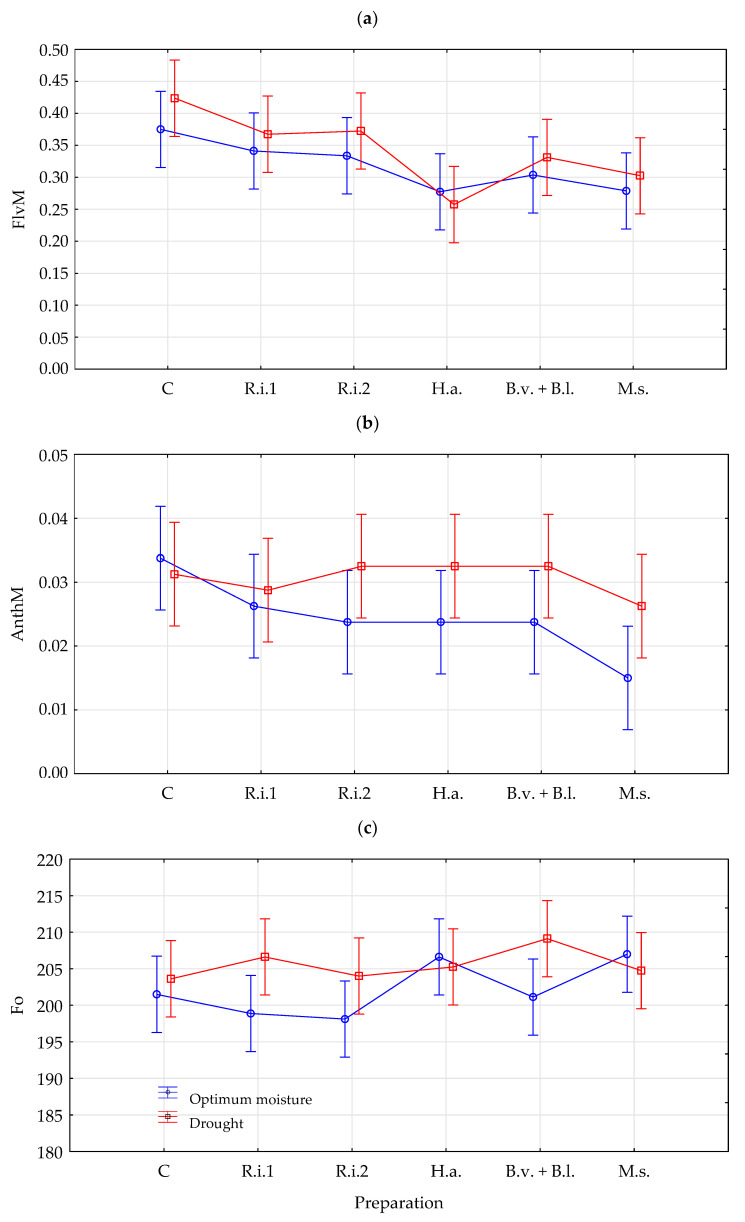
Effect of moisture conditions and preparations on flavonols (FlvM) (**a**), anthocyanins (AnthM) (**b**), and minimal fluorescence (F_0_) (**c**); C—untreated check, R.i.1—*Rhizophagus irregularis* (1), R.i.2—*Rhizophagus irregularis* (2), H.a.—humic acids; B.v. + B.l.—*Bacillus velezensis* + *Bacillus licheniformis*, M.s.—*Methylobacterium symbioticum*.

The soil moisture conditions interaction with the preparations used was statistically important (*p* ≤ 0.05) in the case of FlvM, AnthM content, and F_0_. This indicates that the impact of preparations changed depending on the humidity conditions (Figure 1). Flavonol content values were lower in the conditions of optimal soil moisture and after the use of humic acids, *Bacillus velezensis* + *Bacillus licheniformis*, and *Methylobacterium symbioticum*. In the case of AnthM, higher values of this parameter were found in the conditions of drought, while in combinations with optimal humidity conditions were lower and decreased after the use of biostimulants. The moisture conditions of the soil also affected the effects of preparations expressed by the values of the F_0_ parameter. Higher F_0_ values in the conditions of optimal soil moisture were obtained only after the use of humic acids and *Methylobacterium symbioticum*; however, within individual humidity conditions, no statistically significant differences were found between individual variants of preparations for this parameter within individual soil hydration conditions.

### 2.2. Field Research

In 2022, none of the preparations used had a statistically significant effect on grain yield, 1000 kernel weight, and hectoliter weight. In 2023, the application of *Rhizophagus irregularis* (2), humic acids, and *Bacillus velezensis* + *Bacillus licheniformis* contributed to a statistically significant increase in grain yield by 1.3, 0.8, and 0.9 t ha^−1^, respectively, compared to the control. None of the preparations had a statistically significant effect on 1000 kernel weight and hectoliter weight (Table 3). During both years of this study, the use of biostimulants and soil amendments had no statistically significant effect on the content of protein, oil, and starch in the maize grain (Table 4).

## 3. Discussion

Biostimulants and substances improving soil properties are increasingly used across the world. During research on their effectiveness, it is worth examining their various functions, impact on individual aspects of plant functioning, and their final reflection on the level of plant yield. It is worth noting that individual preparations of this type may differ significantly from each other [44]. Therefore, research should be conducted on their effectiveness on various plants, and in different environmental conditions.

The use of individual substances and microorganisms contributed to the decrease in the content of flavonols. These substances are one of the subclasses of flavonoids [45]. In the studies conducted by Ertani et al. [46], it was found that subjecting maize to salt stress contributed to the increase in the content of flavonoids in the plants, which indicates that these pigments are antioxidants involved in the response to stress. The synthesis of flavonols may occur more effectively under conditions of chlorophyll degradation [47]. In the obtained results, the use of biostimulants allowed for chlorophyll levels to be maintained at a constant level, which could limit the formation of flavonols. The occurrence of stress in plants may also lead to the accumulation of anthocyanins in leaves [48]. These substances contribute to maintaining the homeostasis of reactive oxygen species in plant cells. Water deficiency is one of the factors causing the accumulation of anthocyanins in plants [49]. Such a relationship was also found in the greenhouse experiment—in combinations where plants were subjected to drought stress and biostimulants were used, a higher content of anthocyanins was found than in analogous combinations where plants were optimally watered. Water deficiency in the soil influenced the increase in the level of anthocyanins. In the studies conducted by Franzoni et al. [50], the use of herbicides contributed to the increase in the content of anthocyanins in soybean leaves, and the additional application of biostimulant raw materials had a varied effect on their amount. A deficiency of nutrients may also contribute to the accumulation of these pigments in the leaves [51]. This explains the decrease in their content in plants on which biostimulants were applied in conditions of optimal humidity.

Water shortages in the soil had an impact on a decrease in the height of plants, variable fluorescence, maximum fluorescence, and maximum quantum yield of PSII photochemistry. They also led to an increase in the value of minimal fluorescence. A higher value of F_0_ indicates a reduced efficiency of energy transfer or energy absorption by PSII [52]. Plant growth in drought conditions also led to a decrease in the values of maximum fluorescence and variable fluorescence. Hazrati et al. [53] also found that subjecting plants to stress resulting from water deficiency can lead to a decrease in the values of Fm and Fv parameters. Badr and Brüggemann [54], in their studies, determined the effect of drought on the maximum quantitative yield of PSII photochemistry for different maize genotypes. In the context of all genotypes, lower values of this parameter were noted more in drought conditions than in optimal conditions. Also, in our own studies, higher values of this parameter were found in the variant in which watering was not stopped. These physiological parameters reflect the condition of the plants. The results obtained in greenhouse conditions may also explain the lower yields recorded in 2022, when rainfall levels were much lower than in 2023. The use of biostimulants did not have a statistically significant effect on the chlorophyll fluorescence parameters. In the studies conducted by Radzikowska-Kujawska et al. [55], it was also found that the use of some biostimulants did not affect the selected chlorophyll fluorescence parameters, but had a beneficial effect on the plant growth. Not all biostimulants directly affect the functioning of the photosynthetic apparatus, but ultimately have a beneficial effect on plant growth, which is why it is worth performing various measurements when testing this type of preparation.

In a greenhouse study, it was found that the use of preparations based on *Rhizophagus irregularis*, humic acids, and *Bacillus velezensis* + *Bacillus licheniformis* contributed to a statistically significant increase in plant height. In field conditions, it was checked that substances and microorganisms also affected the yield of maize in 2023. In the first year of field tests, none of the preparations used contributed to a statistically significant increase in maize yield. It is worth noting that, in the 2022 growing season, there were significant water shortages throughout the entire period of crop development. Drought is one of the factors that most limits the level of maize yield in the world [56,57]. Most of the preparations used improve the condition of plants in conditions of water shortage, but extreme water shortage does not allow for them to work effectively. De Clercq et al. [58] also tested various biostimulants in their studies and found that the level of water shortage and its frequency affected their effectiveness. In the year 2023, periodic water shortages were also observed, but the total amount of rainfall was then higher, including at the beginning of the corn vegetation period. During this period, most of the preparations used had a significant impact on the level of maize yield.

In the conducted studies, it is worth paying attention to two combinations in which *Rhizophagus irregularis* was used. These microorganisms were in preparations of different formulations, and they were also characterized by a different concentration of spores. In the case of biostimulants, it is worth noting that the appropriate formulation affects their effectiveness [59]. In the studies conducted by Stoffel et al. [60], the use of arbuscular mycorrhiza contributed to an increase in the yield level of maize. Amerian et al. [61] also proved the positive effect of AM on the physiological parameters of the discussed plant and its delayed wilting. Another preparation used in the own experiment was based on humic acids. The application of this type of biostimulator contributed to an increase in the yield level and plant height of maize grown in greenhouse conditions, as well as a decrease in flavonol levels. In the studies conducted by Rahouma [62], it was also found that the application of this type of substance contributed to an increase in the yield level of maize. Additionally, the results of the studies obtained by Guo et al. [63] indicated that the use of HA together with controlled-release fertilizer results in better nitrogen utilization by the plant and leads to increased yield, while at the same time reducing greenhouse gas emissions. Application of the preparation containing two *Bacillus* strains also had a positive effect on the development of the test plants—the height of plants grown in greenhouse conditions and their yield level in 2023. In the work of Zhang et al. [64], a positive effect of *Bacillus velezensis* on the growth of maize was described. A positive effect of these bacteria was also described, among others, on inorganic phosphorus solubilization, potassium solubilization, and nitrogen fixation determined in laboratory conditions. The use of *Bacillus licheniformis* in the studies of Akhtar et al. [65] contributed to an increase in root mass and improved water use efficiency by maize. The results of the studies obtained by Kulimushi et al. [66] also indicate that the use of *Bacillus* bacteria may have a stimulating effect on plant growth, while at the same time having a positive effect on the health of maize. In our studies, no disease infection of the plants was observed, so this parameter was not considered. The use of a preparation based on *Methylobacterium symbioticum* did not have a statistically significant effect on the growth and yield level of maize grown in greenhouse conditions. In the conducted studies, the experimental factor taken into account was the moisture content of the substrate, and different levels of nitrogen fertilization were not taken into account. In the work of Torres Vera et al. [40], the use of the above-mentioned bacteria with various dosing of fertilizers showed that the application of *Methylobacterium symbioticum* allows for an increase in plant yield at reduced N doses, but it was not noticed at higher amounts of nitrogen. The results obtained in our own studies indicate that at a constant level of fertilization, the application of microorganisms did not contribute to the increase in the above-mentioned features. Nevertheless, it should be noted that the use of given microorganisms will not be reflected in their effectiveness in all conditions [67].

The occurrence of drought contributes, among other things, to disturbances in the transport of nutrients in the plant. The stomata close to limit transpiration, which negatively affects the availability of carbon dioxide, and is used in the photosynthesis process. All of the above-mentioned aspects contribute to limitations in plant growth and their functioning, which could be observed in the described measurements. The use of biostimulants improves plant nutrition, which alleviates many of the negative aspects of drought in plants. Therefore, after the use of test preparations, an improvement in plant growth was observed compared to the control.

## 4. Materials and Methods

### 4.1. Greenhouse Research

The research was conducted in the greenhouse of the Faculty of Agriculture, Horticulture, and Biotechnology of the Poznań University of Life Sciences. The photoperiod in the greenhouse was kept at the constant level of 8 h night/16 h day. Sunlight was automatically supplemented with LED lamps (light source luminous flux 780 lm). Air humidity was maintained at the level 50–80%. The air temperature was in the range 25 ± 2 °C during the day and 20 ± 2 °C during the night.

The experiment used a universal substrate made of frozen peat. Maize grain (Farmoritz variety) was sown into pots with a volume of 1.0 L. Then, the pots with sown grain were placed in water until the soil reached its full water capacity. After this time, all the research objects were watered with an equal amount of water at intervals of two days. After the maize emerged, three plants were left in each pot.

When the plants were in the 2–3 leaves or 4–5 leaves stage, individual preparations were applied. In the control combination, no test preparation was used. In the subsequent variants, the following were used: liquid formulation containing 245 spores gram^−1^ of *Rhizophagus irregularis* (another name *Glomus intraradices*)—called *Rhizophagus* 1 (Poznań University of Life Sciences, Poznań, Poland); solid formulation of *Rhizophagus irregularis*—minimum concentration 270 spores g^−1^—called *Rhizophagus* 2 (Poznań University of Life Sciences, Poznań, Poland); Ferti Agro Humic—total humic extract—85% *w*/*w*, humic acids—70% *w*/*w*, fulvic acids—15% *w*/*w*, K_2_O—9% *w*/*w* (Wialan Technologies, Inc., Tarnów, Poland); plo-N BIO LIDER—*Bacillus velezensis* + *Bacillus licheniformis*—a composition based on bacteria of the genus *Bacillus* at a concentration of 1 × 108 JTK /mL (SCANDAGRA Polska Sp. z o.o., Żołędowo, Poland); BlueN—*Methylobacterium symbioticum* SB23—total bacteria count ≥10^7^ jtk g^−1^ (Corteva Agriscience Poland Sp. z o.o., Warsaw, Poland). The doses of the preparations are given in Table 5. *Rhizophagus irregularis* and humic acids act through the soil, which is why they were applied when the leaves did not cover much of its surface. *Bacillus velezensis* + *Bacillus licheniformis* and *Methylobacterium symbioticum* can be taken up through the leaves, which is why they were applied when the surface of the leaf blades was larger. The treatment was performed using a laboratory sprayer. The spray liquid output was 200 l ha^−1^ at a pressure of 0.2 MPa. Tee Jet 1102 nozzles were used, which were placed 50 cm above the sprayed surface.

Seven days after the application of all the preparations, the objects were divided into two groups—optimal soil moisture and drought. Four repetitions were performed for all treatments, separately for optimal soil hydration and drought. From that time on, plants marked as “drought” were watered with half the amount of water compared to the optimum soil moisture, and after four days, watering of the drought pots was stopped. As the effects of drought on the plants became visible, soil moisture was measured. Soil volume moisture was measured using a probe (ThetaProbe, Royal Eijkelkamp, The Netherlands). The average soil moisture for drought was 4.6% by volume and 29.9% by volume for optimal soil moisture. After obtaining such soil moisture level, measurements were started.

In the greenhouse studies, plant height, chlorophyll fluorescence parameters, and leaf pigment content were measured. The height of all plants was measured. The height of the above-ground part was measured using a ruler with an accuracy of 1 mm. Chlorophyll fluorescence measurements were performed using a modulated fluorometer measures FV/FM (OS5p, Opti-Sciences, Inc., Hudson, NH, USA). The study was performed on the youngest, fully developed leaves. Before the measurement, the leaves were subjected to dark adaptation for 30 min using white darkening clips. According to the instructions for the measuring device, the parameters were set so that the fluorescence signal was in the range of 150–250 units and was stable. The parameters assessed were: F_0_—minimal fluorescence, Fv—variable fluorescence, Fm—maximum fluorescence, Fv/Fm—maximum quantum yield of PSII photochemistry. Chlorophyll, anthocyanin and flavonol contents were determined using an MPM-100 Multi Pigment Meter (Opti-Sciences, Inc., Hudson, NH, USA). For the above parameters, two measurements were performed for each repetition.

### 4.2. Field Research

The field experiment was carried out in the Research and Education Center in Brody, belonging to the Poznań University of Life Sciences (52.435906, 16.290538) in Poland, in 2022 and 2023. Farmoritz variety maize was sown on 28 April 2022, and 5 May 2023, to a depth of 4 cm, with a row spacing of 70 cm. Each plot had an area of 22.5 m^2^. The light soil at this site had a pH level of 6.9. The organic matter content was 1.4%. The experiment was conducted in a randomized design. Four replications were performed for each treatments. In both years, the forecrop for maize was cereals.

The characteristics of precipitation and thermal conditions are presented for decades and for whole months using the Sielianinow’s hydrothermal index calculated according to the following formula:

$$k=\frac{P}{0.1\Sigma t}$$

where:

Σt is sum of air temperatures >0 °C

P is sum of atmospheric precipitation in mm

k is Sielianinow’s hydrothermal index

The results of the calculated Sielianinow’s hydrothermal index were presented for nine classes, in accordance with the following methodology developed by Skowera and Puła [68]:

- Very humid: 2.5 < k ≤ 3.0
- Very dry: 0.4 < k ≤ 0.7
- Slightly humid: 1.6 < k ≤ 2.0
- Slightly dry: 1.0 < k ≤ 1.3
- Optimum: 1.3 < k ≤ 1.6
- Humid: 2.0 < k ≤ 2.5
- Extremely humid: k > 3.0
- Extremely dry: k ≤ 0.4
- Dry: 0.7 < k ≤ 1.0

The values of Sielianinow’s hydrothermal index and rainfall level for the years of the study are presented in Table 6.

Phosphorus and potassium were applied in the autumn of the year preceding the maize sowing at the dose of 50 and 75 kg ha^−1^. In the spring, before sowing the crop, 90 kg N ha^−1^ was applied. In the 6–7 leaves stage of maize, the second dose of nitrogen was applied at 60 kg ha^−1^. Fertilizer doses were adjusted to soil fertility and plant needs. Plant protection products were applied in accordance with the recommendations in force in Poland. *Rhizophagus irregularis* (1), *Rhizophagus irregularis* (2), humic acids, *Bacillus velezensis* + *Bacillus licheniformis*, and *Methylobacterium symbioticum* were applied at the same doses and times, as given in Table 5. The crops were harvested on 17 October 2022 and 16 October 2023, and converted to 15% grain moisture. Grain yields were calculated per area of 1 hectare. Protein, oil, and starch contents of the grain were determined using a Foss Infratec 1241 analyser (FOSS, Hillerød, Denmark).The weight of 1000 grain kernels (TKW) and the mass of 1 hectoliter (HLW) were also tested.

### 4.3. Statistical Analysis

Statistical analysis. A one-way ANOVA analysis of variance was used to verify if there were statistically significant differences in the means between groups in the field experiment. Before analysis, the Shapiro–Wilk test was used to confirm that the distribution of results in the analyzed data was close to normal. The assumption of homogeneity of variance was evaluated using Levene’s test. If the results indicated that the assumption of homogeneity of variance was not fulfilled, Welch’s test was applied, which introduced corrections for unequal variances. The effect of a given factor on the variables, which in the experiment were the preparations used, was analyzed using an ANOVA analysis of variance. The null hypothesis assumed that the means in all groups were equal, and that there were no statistically significant differences among them. In instances where the results of the ANOVA test were statistically significant, the null hypothesis was rejected and it was presumed that the value in at least one of the groups differed from the others. A Tukey HSD post hoc test was conducted to determine which group averages were statistically significantly different from one another. In the greenhouse experiment, two factors were analyzed: moisture conditions (optimum and drought) and preparations (control and 5 preparations) A two-factor analysis of variance was employed to identify the potential impact of the two factors on the traits. The outcomes of the analysis are presented separately for each of the two factors. The cases where an interaction between moisture conditions and the preparations used were confirmed are presented as graphs.

## 5. Conclusions

Maize is one of the most important crops in the world, which is why research on counteracting drought in this crop is important from the point of view of agricultural science and practice. The solution that can reduce the adverse impact of environmental factors is the use of appropriate biostimulants and soil amendments. The conducted studies used preparations containing *Rhizophagus irregularis*, humic acids, *Bacillus velezensis* + *Bacillus licheniformis*, and *Methylobacterium symbioticum*. In greenhouse conditions, it was shown that drought contributed to the deterioration of the condition and growth of plants. Most of the parameters used had a beneficial effect on the height of the plants, and also contributed to a decrease in the value of anthocyanins and flavonols. In field studies, the effect of biostimulants and soil amendments depended on the conditions prevailing in a given growing season. The best effects in terms of yield were noted after the use of one of the formulations *Rhizophagus irregularis*, humic acids, and *Bacillus velezensis* + *Bacillus licheniformis*. As experiments have shown, the effects of this type of preparations are worth testing under various conditions.

## Figures and Tables

**Table 1:** Impact of moisture conditions of soil on parameters assessed in greenhouse conditions.

No.	Feature	Moisture Conditions	
		Optimum	Drought
1.	Height	80.6 a	75.9 b
2.	ChlM	0.57 a	0.60 a
3.	FlvM	0.32 a	0.34 a
4.	AnthM	0.024 b	0.031 a
5.	F_0_	202.2 b	205.6 a
6.	Fv	843.8 a	798.4 b
7.	Fm	1046.0 a	1003.9 b
8.	Fv/Fm	0.806 a	0.795 b

Means followed by the same letter in the line do not differ according to Tukey’s HSD test at =0.05; height—cm; ChlM, FlvM, AnthM—absolute unit; F_0_, Fv, Fm, Fv/Fm—not-nominated units; ChlM—chlorophyll content, FlvM—flavonol content, AnthM—anthocyanin content, F_0_—minimal fluorescence of dark-adapted state, Fv—variable fluorescence, Fm—maximum fluorescence of dark-adapted state, Fv/Fm—maximum quantum yield of PSII photochemistry.

**Table 2:** Impact of biostimulants and soil amendments on parameters assessed in greenhouse conditions.

No.	Preparation	Height	ChlM	FlvM	AnthM	F_0_	Fv	Fm	Fv/Fm
1.	Untreated check	75.0 b	0.54 a	0.40 a	0.033 a	202.6 a	809.4 a	1012 a	0.799 a
2.	*Rhizophagus irregularis* (1)	80.2 a	0.60 a	0.35 ab	0.028 ab	202.8 a	822.1 a	1025 a	0.801 a
3.	*Rhizophagus irregularis* (2)	79.3 a	0.61 a	0.35 ab	0.028 ab	201.1 a	815.2 a	1016 a	0.802 a
4.	Humic acids	79.4 a	0.58 a	0.27 c	0.028 ab	205.9 a	831.8 a	1038 a	0.801 a
5.	*Bacillus velezensis* + *Bacillus licheniformis*	80.7 a	0.60 a	0.32 abc	0.028 ab	205.1 a	825.4 a	1031 a	0.800 a
6.	*Methylobacterium symbioticum*	75.2 b	0.59 a	0.29 bc	0.021 b	205.9 a	822.8 a	1029 a	0.799 a

Means followed by the same letter in the line do not differ according to Tukey’s HSD test at =0.05; height—cm; ChlM, FlvM, AnthM—absolute unit; F_0_, Fv, Fm, Fv/Fm—not-nominated units, ChlM—chlorophyll content, FlvM—flavonol content, AnthM—anthocyanin content, F0—minimal fluorescence of dark-adapted state, Fv—variable fluorescence, Fm—maximum fluorescence of dark-adapted state, Fv/Fm—maximum quantum yield of PSII photochemistry.

**Table 3:** Impact of biostimulants and soil amendments on grain yield, 1000 kernel weight, and hectoliter weight.

No.	Biostimulant	Yield [t ha^−1^]				TKW [g]				HLW [kg 100 L^−1^]			
		2022	SE	2023	SE	2022	SE	2023	SE	2022	SE	2023	SE
1.	Untreated check	9.2 a	0.38	12.4 c	0.14	283.7 a	3.36	379.6 a	1.20	66.7 a	0.31	69.1 a	0.68
2.	*Rhizophagus irregularis* (1)	9.5 a	0.15	12.6 c	0.08	292.6 a	5.64	376.4 a	5.14	67.0 a	0.05	70.2 a	0.77
3.	*Rhizophagus irregularis* (2)	9.2 a	0.15	13.7 a	0.10	287.4 a	2.99	376.5 a	5.16	67.0 a	0.36	71.2 a	0.35
4.	Humic acids	9.4 a	0.14	13.2 ab	0.17	285.9 a	1.99	373.2 a	3.77	67.0 a	0.31	71.2 a	0.54
5.	*Bacillus velezensis* + *Bacillus licheniformis*	9.9 a	0.15	13.3 a	0.07	295.4 a	2.56	376.7 a	2.31	66.6 a	0.15	70.5 a	0.86
6.	*Methylobacterium symbioticum*	9.9 a	0.40	12.7 bc	0.11	296.3 a	3.08	374.3 a	2.34	66.1 a	0.31	70.1 a	0.74

TKW: 1000 kernel weight; HLW: hectoliter weight; SE: standard error. Mean followed by the same letter in the column do not differ according to Tukey’s HSD test at =0.05.

**Table 4:** Impact of biostimulants and soil amendments on proteins, oil, and starch in maize grain.

No.	Biostimulant	Content %											
		Protein				Oil				Starch			
		2022	SE	2023	SE	2022	SE	2023	SE	2022	SE	2023	SE
1.	Untreated check	11.8 a	0.23	12.1 a	0.19	3.5 a	0.06	3.6 a	0.06	69.1 a	0.19	70.2 a	0.12
2.	*Rhizophagus irregularis* (1)	12.3 a	0.11	12.3 a	0.36	3.6 a	0.04	3.6 a	0.04	68.9 a	0.28	70.0 a	0.05
3.	*Rhizophagus irregularis* (2)	11.5 a	0.30	11.7 a	0.28	3.5 a	0.03	3.6 a	0.03	69.4 a	0.29	70.3 a	0.19
4.	Humic acids	11.7 a	0.15	11.9 a	0.19	3.6 a	0.02	3.6 a	0.01	69.0 a	0.30	70.4 a	0.10
5.	*Bacillus velezensis* + *Bacillus licheniformis*	12.0 a	0.53	12.1 a	0.49	3.6 a	0.02	3.7 a	0.03	68.7 a	0.26	70.1 a	0.21
6.	*Methylobacterium symbioticum*	11.4 a	0.19	11.0 a	0.33	3.5 a	0.02	3.6 a	0.04	69.4 a	0.17	70.4 a	0.13

SE: standard error. Mean followed by the same letter in the column do not differ according to Tukey’s HSD test at =0.05.

**Table 5:** Scheme of the conducted research.

No.	Preparation	Abbreviation	Dose (kg ha^−1^)	Maize Development Stage
1	Untreated check	C	-	-
2	*Rhizophagus irregularis* (1)	R.i.1	0.25 L	2–3 leaves
3	*Rhizophagus irregularis* (2)	R.i.2	0.2 kg	2–3 leaves
4	Humic acids	H.a.	1.0 kg	2–3 leaves
5	*Bacillus velezensis* + *Bacillus licheniformis*	B.v. + B.l.	0.5 L	4–5 leaves
6	*Methylobacterium symbioticum*	M.s.	0.333 kg	4–5 leaves

**Table 6:** Sielianinow’s hydrothermal index (S. index) and rainfall (mm) for 2022 and 2023.

Months		Decade of the Month			Average for the Month
		I	II	III	
	2022				
April	S. index	4.2	1.6	0.0	1.4
	rainfall	20.6	12.1	0.0	32.7
May	S. index	0.6	0.7	0.6	0.6
	rainfall	8.2	11.4	9.2	28.8
June	S. index	0.9	1.7	0.4	0.9
	rainfall	14.9	31.7	9.4	56.0
July	S. index	0.5	0.5	0.4	0.5
	rainfall	8.6	9.5	9.3	27.4
August	S. index	0.0	0.7	1.2	0.6
	rainfall	0.0	17.3	24.8	42.1
September	S. index	1.1	0.7	0.5	0.8
	rainfall	17.8	9.4	5.5	32.7
October	S. index	0.3	1.1	0.7	0.7
	rainfall	3.9	12.0	10.1	26.0
Total rainfall					245.7
	2023				
April	S. index	2.1	4.6	0.5	2.3
	rainfall	9.6	40	4.7	54.3
May	S. index	1.7	1.0	0.6	1.0
	rainfall	18.6	13.2	9.5	41.3
June	S. index	0.0	0.3	1.4	0.6
	rainfall	0.0	6.1	28.7	34.8
July	S. index	0.4	0.5	2.0	1.0
	rainfall	8.0	11.6	37.0	56.6
August	S. index	5.6	0.9	2.2	2.7
	rainfall	95.6	19.7	39.9	155.2
September	S. index	0.0	0.3	0.6	0.2
	rainfall	0.0	5.2	6.7	11.9
October	S. index	1.6	1.4	5.3	2.8
	rainfall	20.8	14.3	60.1	95.2
Total rainfall					449.3

## Data Availability

The original contributions presented in this study are included in the article. Further inquiries can be directed to the corresponding author.

## Abbreviations

F_0_	minimal fluorescence of dark-adapted state
Fv	variable fluorescence
Fm	maximum fluorescence of dark-adapted state
Fv/Fm	maximum quantum yield of PSII photochemistry
ChlM	chlorophyll content
FlvM	flavonol content
AnthM	anthocyanin content
TKW	1000 kernel weight
HLW	hectoliter weight
AM	arbuscular mycorrhiza

## References

[1] Liliane T., Mutengwa C. (2020). Factors Affecting Yield of Crops, Agronomy—Climate Change & Food Security.

[2] Idziak R., Waligóra H., Szuba V. (2022). The influence of agronomical and chemical weed control on weeds of corn. J. Plant Prot. Res..

[3] Basso B., Ritchie J. (2014). Temperature and drought effects on maize yield. Nat. Clim. Chang..

[4] Caverzan A., Casassola A., Brammer S.P., Shanker A.K., Shanker C. (2016). Reactive oxygen species and antioxidant enzymes involved in plant tolerance to stress. Abiotic and Biotic Stress in Plants-Recent Advances and Future Perspectives.

[5] Sun Z., Yang R., Wang J., Zhou P., Gong Y., Gao F., Wang C. (2024). Effects of Nutrient Deficiency on Crop Yield and Soil Nutrients Under Winter Wheat–Summer Maize Rotation System in the North China Plain. Agronomy.

[6] Bhattacharyya S.S., Ros G.H., Furtak K., Iqbal H.M.N., Parra-Saldìvar R. (2022). Soil carbon sequestration—An interplay between soil microbial community and soil organic matter dynamics. Sci. Total Environ..

[7] Du Jardin P. (2015). Plant biostimulants: Definition, concept, main categories and regulation. Sci. Hortic..

[8] Ali S., Jan A., Sohail A., Khan A., Khan M.I., Zhang J., Daur I. (2018). Soil amendments strategies to improve water-use efficiency and productivity of maize under different irrigation conditions. Agric. Water Manag..

[9] Ranum P., Pablo J., Peña R., Garcia-Casal M.N. (2014). Global maize production, utilization, and consumption. Ann. N. Y. Acad. Sci..

[10] Patil H., Athalye A. (2023). Valorization of Corn Husk Waste for Textile Applications. J. Nat. Fibers.

[11] USDA Foreign Agricultural Service Production–Corn. https://www.fas.usda.gov/data/production/commodity/0440000.

[12] Ocwa A., Mohammed S., Mousavi S.M.N., Illés Á., Bojtor C., Ragán P., Rátonyi T., Harsányi H. (2024). Maize grain yield and quality improvement through biostimulant application: A systematic review. J. Soil Sci. Plant Nutr..

[13] Onyeaka H.N., Akinsemolu A.A., Siyanbola K.F., Adetunji V.A. (2024). Green Microbe Profile: *Rhizophagus intraradices*—A Review of Benevolent Fungi Promoting Plant Health and Sustainability. Microbiol. Res..

[14] Gianinazzi-pearson V. (1996). Plant cell responses to arbuscular mycorrhizal fungi: Getting to the roots of the symbiosis. Plant Cell.

[15] Bhantana P., Rana M.S., Sun X.-c., Moussa M.G., Saleem M.H., Syaifudin M., Shah A., Poudel A., Pun A.B., Bhat M.A. (2021). Arbuscular mycorrhizal fungi and its major role in plant growth, zinc nutrition, phosphorous regulation and phytoremediation. Symbiosis.

[16] Khaliq A., Perveen S., Alamer K.H., Zia Ul Haq M., Rafique Z., Alsudays I.M., Althobaiti A.T., Saleh M.A., Hussain S., Attia H. (2022). Arbuscular Mycorrhizal Fungi Symbiosis to Enhance Plant–Soil Interaction. Sustainability.

[17] Vlček V., Pohanka M. (2020). Glomalin—An interesting protein part of the soil organic matter. Soil Water Res..

[18] Öpik M., Moora M., Liira J., Zobel M. (2006). Composition of root-colonizing arbuscular mycorrhizal fungal communities in different ecosystems around the globe. J. Ecol..

[19] Delaeter M., Magnin-Robert M., Randoux B., Lounès-Hadj Sahraoui A. (2024). Arbuscular Mycorrhizal Fungi as Biostimulant and Biocontrol Agents: A Review. Microorganisms.

[20] Pettit R.E. Organic Matter, Humus, Humate, Humic acid, Fulvic Acid and Humin. Wonderful World Humus Carbon 2006. https://www.semanticscholar.org/paper/ORGANIC-MATTER-,-HUMUS-,-HUMATE-,-HUMIC-ACID-,-ACID-Pettit/bd2e61da484c14d9325d16024360a118c4809ba1.

[21] Ampong K., Thilakaranthna M.S., Gorim L.Y. (2022). Understanding the role of humic acids on crop performance and soil health. Front. Agron..

[22] Sible C.N., Seebauer J.R., Below F.E. (2021). Plant Biostimulants: A Categorical Review, Their Implications for Row Crop Production, and Relation to Soil Health Indicators. Agronomy.

[23] Berbara R.L.L., García A.C. (2014). Humic substances and plant defense metabolism. Physiological Mechanisms and Adaptation Strategies in Plants Under Changing Environment.

[24] Ouni Y., Ghnaya T., Montemurro F., Abdelly C., Lakhdar A. (2014). The role of humic substances in mitigating the harmful effects of soil salinity and improve plant productivity. Int. J. Plant Prod..

[25] Calvo P., Nelson L., Kloepper J.W. (2014). Agricultural uses of plant biostimulants. Plant Soil.

[26] Kaya C., Şenbayram M., Akram N.A., Ashraf M., Alyemeni M.N., Ahmad P. (2020). Sulfur-enriched leonardite and humic acid soil amendments enhance tolerance to drought and phosphorus deficiency stress in maize (*Zea mays* L.). Sci. Rep..

[27] Canellas L.P., Canellas N.O., Irineu L.E.S.D.S., Olivares F.L., Piccolo A. (2020). Plant chemical priming by humic acids. Chem. Biol. Technol. Agric..

[28] Bhatt P., Singh V.K. (2022). Efect of humic acid on soil properties and crop production. A review. Indian J. Agricult. Sci..

[29] Khan A.R., Mustafa A., Hyder S., Valipour M., Rizvi Z.F., Gondal A.S., Yousuf Z., Iqbal R., Daraz U. (2022). *Bacillus* spp. as Bioagents: Uses and Application for Sustainable Agriculture. Biology.

[30] Etesami H., Jeong B.R., Glick B.R. (2023). Potential use of *Bacillus* spp. as an effective biostimulant against abiotic stresses in crops—A review. Curr. Res. Biotechnol..

[31] Fan B., Wang C., Song X., Ding X., Wu L., Wu H., Gao X., Borriss R. (2018). *Bacillus velezensis* FZB42 in 2018: The Gram-Positive Model Strain for Plant Growth Promotion and Biocontrol. Front. Microbiol..

[32] Keshmirshekan A., de Souza Mesquita L.M., Ventura S.P.M. (2024). Biocontrol manufacturing and agricultural applications of *Bacillus velezensis*. Trends Biotechnol..

[33] Jang S., Choi S.-K., Zhang H., Zhang S., Ryu C.-M., Kloepper J.W. (2023). History of a model plant growth-promoting rhizobacterium, *Bacillus velezensis* GB03: From isolation to commercialization. Front. Plant Sci..

[34] Gutierrez-Manero F.J., Ramos-Solano B., Probanza A., Mehouachi J.R., Tadeo F., Talon M. (2001). The plant-growth-promoting rhizobacteria *Bacillus pumilus* and *Bacillus licheniformis* produce high amounts of physiologically active gibberellins. Physiol. Plant.

[35] Ni S., Wu Y., Zhu N., Leng F., Wang Y. (2024). *Bacillus licheniformis*YB06: A Rhizosphere–Genome-Wide Analysis and Plant Growth-Promoting Analysis of a Plant Growth-Promoting Rhizobacterium Isolated from Codonopsis pilosula. Microorganisms.

[36] Omer Z.S., Tombolini R., Gerhardson B. (2004). Plant colonization by pink-pigmented facultative methylotrophic bacteria (PPFMs). FEMS Microbiol. Ecol..

[37] Dourado M.N., Camargo Neves A.A., Santos D.S., Araújo W.L. (2015). Biotechnological and agronomic potential of endophytic pink-pigmented methylotrophic *Methylobacterium* spp.. BioMed Res. Int..

[38] Kutschera U. (2007). Plant-associated methylobacteria as co-evolved phytosymbionts: A hypothesis. Plant Signal. Behav..

[39] Pascual J.A., Ros M., Martínez J., Carmona F., Bernabé A., Torres R., Lucena T., Aznar R., Arahal D.R., Fernández F. (2020). *Methylobacterium symbioticum* sp. nov., a new species isolated from spores of *Glomus iranicum* var. tenuihypharum. Curr. Microbiol..

[40] Torres Vera R., Bernabé García A.J., Carmona Álvarez F.J., Martínez Ruiz J., Fernández Martín F. (2024). Application and effectiveness of *Methylobacterium symbioticum* as a biological inoculant in maize and strawberry crops. Folia Microbiol..

[41] Amato G., Cardone L., Cicco N., Denora M., Perniola M., Casiello D., De Martino L., De Feo V., Candido V. (2024). Morphological traits, yield, antioxidant activity and essential oil composition of oregano as affected by biostimulant foliar applications. Ind. Crops Prod..

[42] Martínez-Dalmau J., Berbel J., Ordóñez-Fernández R. (2021). Nitrogen Fertilization. A Review of the Risks Associated with the Inefficiency of Its Use and Policy Responses. Sustainability.

[43] Lin N., Wang H., Moscardelli L., Shuster M. (2024). The dual role of low-carbon ammonia in climate-smart farming and energy transition. J. Clean. Prod..

[44] Li J., Van Gerrewey T., Geelen D. (2022). A Meta-Analysis of Biostimulant Yield Effectiveness in Field Trials. Front. Plant Sci..

[45] Hui C., Qi X., Qianyong Z., Xiaoli P., Jundong Z., Mantian M. (2013). Flavonoids, flavonoid subclasses and breast cancer risk: A meta-analysis of epidemiologic studies. PLoS ONE.

[46] Ertani A., Schiavon M., Muscolo A., Nardi S. (2013). Alfalfa plant-derived biostimulant stimulate short-term growth of salt stressed *Zea mays* L. plants. Plant Soil.

[47] Mattila H., Valev D., Havurinne V., Khorobrykh S., Virtanen O., Antinluoma M., Mishra K.B., Tyystjärvi E. (2018). Degradation of chlorophyll and synthesis of flavonols during autumn senescence—The story told by individual leaves. AoB Plants.

[48] Li Z., Ahammed G.J. (2023). Hormonal regulation of anthocyanin biosynthesis for improved stress tolerance in plants. Plant Physiol. Biochem..

[49] Shi L., Li X., Fu Y., Li C. (2023). Environmental Stimuli and Phytohormones in Anthocyanin Biosynthesis: A Comprehensive Review. Int. J. Mol. Sci..

[50] Franzoni G., Bulgari R., Florio F.E., Gozio E., Villa D., Cocetta G., Ferrante A. (2023). Effect of biostimulant raw materials on soybean (*Glycine max*) crop, when applied alone or in combination with herbicides. Front. Agron..

[51] Jezek M., Allan A.C., Jones J.J., Geilfus C.-M. (2023). Why Do Plants Blush When They Are Hungry?. New Phytol..

[52] Kalaji M.H., Łoboda T. (2010). Fluorescencja Chlorofilu w Badaniach Stanu Fizjologicznego ro´slin (The Chlorophyll Fluorescence in the Research of the Physiological State of Plants).

[53] Hazrati S., Tahmasebi-Sarvestani Z., Modarres-Sanavy S.A.M., Mokhtassi-Bidgoli A., Nicola S. (2016). Effects of water stress and light intensity on chlorophyll fluorescence parameters and pigments of *Aloe vera* L.. Plant Physiol. Biochem..

[54] Badr A., Brüggemann W. (2020). Comparative Analysis of Drought Stress Response of Maize Genotypes Using Chlorophyll Fluorescence Measurements and Leaf Relative Water Content. Photosynthetica.

[55] Radzikowska-Kujawska D., John P., Piechota T., Nowicki M., Kowalczewski P.Ł. (2023). Response of Winter Wheat to Selected Biostimulants under Drought Conditions. Agriculture.

[56] Aslam M., Maqbool M.A., Cengiz R. (2015). Drought Stress in Maize (Zea mays L.) Effects, Resistance Mechanisms, Global Achievements and Biological Strategies for Improvement.

[57] Daryanto S., Wang L., Jacinthe P.A. (2016). Global Synthesis of Drought Effects on Maize and Wheat Production. PLoS ONE.

[58] De Clercq P., Pauwels E., Top S., Steppe K., Van Labeke M.-C. (2023). Effect of Seaweed-Based Biostimulants on Growth and Development of *Hydrangea paniculate* under Continuous or Periodic Drought Stress. Horticulturae.

[59] Kumar H.D., Aloke P. (2020). Role of biostimulant formulations in crop production: An overview. Int. J. Appl. Res. Vet. M..

[60] Stoffel S.C.G., Soares C.R.F.S., Meyer E., Lovato P.E., Gianchini A.J. (2020). Yield increase of corn inoculated with a commercial arbuscular mycorrhizal inoculant in Brazil. Cienc. Rural.

[61] Amerian M.R., Stewart W.S., Griffiths H. (2001). Effect of two species of arbuscular mycorrhizal fungi on growth, assimilation and leaf water relations in maize (*Zea mays*). Asp. Appl. Biol..

[62] Rahouma M.A.A. (2021). Maize growth and yield response to different rates of humic acid and zinc. Alex. Sci. Exch. J..

[63] Guo Y., Ma Z., Ren B., Zhao B., Liu P., Zhang J. (2022). Effects of Humic Acid Added to Controlled-Release Fertilizer on Summer Maize Yield, Nitrogen Use Efficiency and Greenhouse Gas Emission. Agriculture.

[64] Zhang Y., Zhang N., Bi X., Bi T., Baloch F.B., Miao J., Zeng N., Li B., An Y. (2024). Growth promotion on maize and whole-genome sequence analysis of *Bacillus velezensis* D103. Microbiol. Spectr..

[65] Akhtar S.S., Amby D.B., Hegelund J.N., Fimognari L., Großkinsky D.K., Westergaard J.C., Müller R., Moelbak L., Liu F., Roitsch T. (2020). *Bacillus licheniformis* FMCH001 increases water use efficiency via growth stimulation in both normal and drought conditions. Front. Plant Sci..

[66] Kulimushi P.Z., Basime G.C., Nachigera G.M., Thonart P., Ongena M. (2018). Efficacy of *Bacillus amyloliquefaciens* as biocontrol agent to fight fungal diseases of maize under tropical climates: From lab to field assays in south Kivu. Environ. Sci. Pollut. Res..

[67] Rodrigues M.Â., Correia C.M., Arrobas M. (2024). The Application of a Foliar Spray Containing *Methylobacterium symbioticum* Had a Limited Effect on Crop Yield and Nitrogen Recovery in Field and Pot-Grown Maize. Plants.

[68] Skowera B., Puła J. (2004). Skrajne warunki pluwiotermiczne w okresie wiosennym na obszarze Polski w latach 1971–2000. (Pluviometric extreme conditions in spring season in Poland in the years 1971–2000). Acta Agrophys..

